# Associations of Mediterranean Diet, Psychological Wellbeing and Media Pressure on Physical Complexion and Effect of Weekly Physical Activity Engagement in Higher Education

**DOI:** 10.3390/ejihpe13090116

**Published:** 2023-08-25

**Authors:** Eduardo Melguizo-Ibáñez, Gabriel González-Valero, José Luis Ubago-Jiménez, José Manuel Alonso-Vargas, Pilar Puertas-Molero

**Affiliations:** Department of Didactics of Musical, Plastic and Corporal Expression, University of Granada (Spain), Campus de Cartuja, 18071 Granada, Spain; emelguizo@ugr.es (E.M.-I.); ggvalero@ugr.es (G.G.-V.); jlubago@ugr.es (J.L.U.-J.); pilarpuertas@correo.ugr.es (P.P.-M.)

**Keywords:** higher education, wellbeing, Mediterranean diet, physical activity, physical build

## Abstract

Nowadays, the media has the power to encourage active and healthy lifestyles; however, it can have a negative impact on body image and psychological wellbeing. The present research aims to analyze Mediterranean diet adherence, media pressure, slim and athletic build ideals and psychological wellbeing as a function of weekly physical activity engagement. A further aim is to examine the effect of Mediterranean diet adherence, media pressure and psychological wellbeing on the perceived pressure to have an athletic and slim build. The present non-experimental study included a sample of 634 university students. Validated instruments adapted by the scientific community were used for data collection. Gathered data reveal that young people who engage in more than 300 min of physical activity per week are more likely to adhere to a Mediterranean diet, have better psychological wellbeing and feel more pressure to obtain an athletic build. In conclusion, weekly physical activity engagement impacts the variables under study.

## 1. Introduction

Adolescence is a fundamental period of human development [1]. During this stage, behaviors emerge that will determine whether or not the future adult population are physically active [2]. Despite the importance of this developmental stage for encouraging active lifestyles in early adulthood, the adolescent population has been observed to be increasingly sedentary [3].

A sedentary lifestyle has been observed to lead to a gradual worsening of health status [4]. Consequently, negative health outcomes such as increased blood pressure and higher cardiovascular disease risk have been observed [4]. In contrast, it has been shown that more time spent engaged in physical activity helps to improve physical and mental health [5]. Similarly, criteria such as physical exercise intensity and weekly physical activity time must be considered to achieve health improvements [6]. In terms of the intensity of weekly sport engagement, physical activity at a moderate-vigorous intensity each week helps to reduce the risk of suffering from cardiovascular diseases and leads to better physiological and mental outcomes [7]. Light physical activity engagement has also been observed to help improve quality of life and prevent various types of cardiovascular diseases and different types of cancer [6]. To this end, the World Health Organization [6] has established a series of criteria for weekly physical activity engagement. These recommendations state that 150–300 min of aerobic physical activity should be undertaken in the adult population and an average of 60 min per day should be performed by children and adolescents. Currently, research highlights that a high number of adults do not meet the physical activity recommendations outlined by the WHO [8,9].

At a biochemical level, regular physical activity is observed to act as a stressor on the body [10]. When faced with this stress, body organs adapt their function to maintain the body’s homeostasis [10]. One of the systems involved in this process is the neuroendocrine system [10]. The central nervous system, together with the endocrine system, through the hypothalamic-pituitary-pituitary gland effector axis, emits a response that varies according to the intention, volume, duration and type of physical activity [10]. The endocrine system response to physical exercise is mediated by the secretion of different hormones that enter the circulatory system in order to maintain homeostasis [10]. The synthesis of these mediators and their subsequent action will depend on the duration of exercise [10]:

- Immediate hormonal response (the first 30 min of exercise): Central stimulation at the level of the hypothalamus generates an adrenal sympathetic stimulation with increased production of catecholamines (adrenaline and norepinephrine). This inhibits insulin synthesis and stimulates renin production, with these hormones exerting their subsequent effects on all the cells of the organism [10].
- Middle response (after 30 to 60 min of exercise): pituitary hormones such as GH, adrenocorticotropin (ACTH), prolactin (PRL), antidiuretic hormone or vasopressin (ADH) and thyrotropin (TSH) are secreted [10].
- Late response (more than 60 min of exercise): parasympathetic stimulation induces ACTH production and, consequently, the release of glucagon, somatostatin, secretin, vasoactive intestinal polypeptide and pancreatic polypeptide [10].

The release of these hormones is beneficial for the body and has a positive impact on the wellbeing and mental state of young people. Sedentary individuals also report lower levels of wellbeing than those who are physically active [10].

Another issue that is very much present during adolescence and early adulthood is the preference for high-calorie foods [11]. The dietary pattern known as the Mediterranean diet was conceived from within the Mediterranean setting. This is characterized by the intake of foods inherent to the Mediterranean region, such as nuts, vegetables, fruits, legumes and oily fish [12]. Further, this dietary pattern is characterized by a low intake of saturated fat and red meat [12]. High Mediterranean diet adherence has been shown to have health benefits. In this case, good adherence to this dietary pattern has been shown to be associated with a 10% reduction in cardiovascular morbidity and mortality and improvements in the lymphatic system [13]. In addition, mental health benefits have been observed, with greater life satisfaction and control over psychological and emotional wellbeing having been reported [13].

Trends emerge within the young adult population towards caring about body image [14]. Previously conducted studies have highlighted this trend, stating that young people pay greater attention to their diet and strive to reduce sedentary time as a means to obtaining a better physical complexion [14]. Research examining this tendency has revealed that, in many cases, obsessive concern for one’s body image may stem from self-imposed pressure from the internal or external environment [14,15]. It has been observed that external pressure often comes from different social networks or the media [15]. In the youth population, a very high consumption of media content has been observed [16]. There are many areas within the media that focus on taking care of body image [16]. Research argues that the transmission of such messages in the media and on social networks can lead to body dissatisfaction in young people [16]. When such negative perceptions persist over time, they can directly affect young people’s psychological wellbeing [16].

Throughout the 21st century, a large number of studies have been carried out focusing on the wellbeing of young people [17]. Initially, wellbeing was studied from two perspectives [18]. On esuch perspective focused on the hedonic realm, related with happiness, whilst another perspective concerned the eudemonic dimension, which is associated with the functional potential of human beings [18]. Nowadays, wellbeing research focuses on the development of different capabilities related to personal growth [19]. Wellbeing has also been found to act as an indicator of optimal personal functioning [20]. In the latter perspective, positive relationships have been observed between wellbeing and leading an active and healthy lifestyle [19]. In addition, better wellbeing has been observed when young people report an acceptance of their body condition [20]. All of that discussed above introduces a number of issues related with this topic of interest. The following research hypotheses are then proposed:

**Hypothesis 1.**  *Participants who engage in more than 300 min of physical activity per week will show less media pressure.*

**Hypothesis 2.**  *Participants who engage in more than 300 min of physical activity will show a higher level of psychological well-being and Mediterranean diet adherence.*

**Hypothesis 3.**  *Less active participants will show a greater effect of adherence to the Mediterranean diet on athletic build.*

**Hypothesis 4.**  *For more active participants, a positive effect of Mediterranean diet adherence on slim build ideals will be shown.*

In view of this, the present study has the following aim:

Analyze the influence of Mediterranean diet adherence, media pressure, pressure for a slim and athletic build and psychological wellbeing as a function of weekly physical activity engagement.

## 2. Materials and Methods

### 2.1. Participants and Study Design

The present study follows a descriptive, comparative, exploratory and non-experimental design. The sample consisted of 634 university students. With regard to inclusion criteria, participants were required to be undertaking a university degree at the time of data collection. With regard to gender distribution, 352 participants (55.5%) were male and 282 (44.5%) were female. Participants were aged between 18 and 35 years (25.78 ± 6.27 years).

### 2.2. Instruments

**Ad hoc questionnaire:** A questionnaire was designed specifically for the purpose of the present research. This was used to collect socio-demographic and sporting variables. In terms of socio-demographics, data related to sex (male/female) and age were collected. Data on the time spent engaged in physical activity each week was also collected. Weekly physical activity recommendations of the World Health Organization [6] were used for this purpose. Participants were categorized into the following groups based on their responses: less than 150 min, between 150 and 300 min and more than 300 min [21].

**Sociocultural Attitudes Towards Appearance Questtionnaire-4 (SATAQ-4) [22]:** This instrument has been used to collect data related to media pressure. It has also been used to collect data related to pressure to maintain a slim and athletic build. Due to characteristics of the present study population, the Spanish adapted version was used [23]. Reliability of the instrument was examined via Cronbach alpha. Outcomes were α = 0.889, α = 0.904 and α = 0.900 for the Mass Media Pressure, Athletic Complexion and Thin Complexion subscales, respectively. 

**Psychological Wellbeing Scale (PWBS) [24]:** This instrument has been used to collect data related to psychological wellbeing. The version adapted for use with Spanish populations was used [25]. The questionnaire consists of a total of 38 items. These are evaluated along a Likert scale where 1 describes total disagreement and 6 describes total agreement. For this instrument, a value of α = 0.957 was produced. 

**Predimed Questionnaire [26]:** Specifically, the version of Álvarez-Alvárez et al. [27] was used. This instrument has been used to collect data related to Mediterranean diet adherence in adult populations. It comprises 14 items that determine the level to which the Mediterranean diet is adhered to. A Cronbach alpha of α = 0.803 was produced. 

### 2.3. Procedure

**Figure 1:**
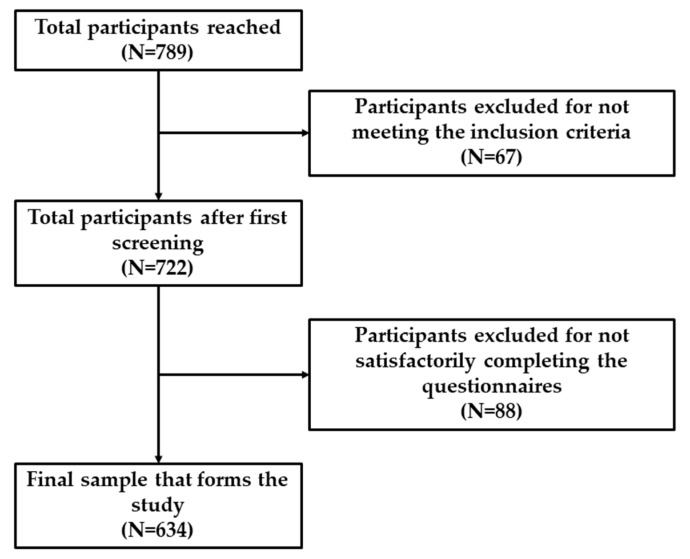
Sample screening process.

Research objectives were defined prior to the initiation of data collection. Data were collected virtually through the Google Forms platform. This made it possible to reach a larger number of university students. Likewise, the questionnaire was shared through various specialized websites by university faculty staff at various Spanish universities. A total of 789 responses were collected, although 155 responses had to be discarded. Inclusion criteria required participants to be undertaking a university degree at a public university in Spain. The final sample consisted of 634 students from different Spanish universities. Figure 1 presents a flow diagram of the sample screening process. 

Prior to completing the questionnaire, all participants provided their informed written consent. Participants were also assured that data would be treated anonymously and used exclusively for scientific purposes. Finally, present research adhered to the ethical principles set out in the Declaration of Helsinki and was supervised by an ethics committee (2966/CEIH/2022).

### 2.4. Data Analysis

First, the distribution of gathered data was examined to determine whether assumptions of normality had been met and determine the statistical tests to be carried out. For this purpose, the Kolmogorov–Smirnov test with Lilliefors correction was conducted. Homoscedasticity was also measured using the Levene test. Test outcomes revealed that data followed a non-normal distribution. Analysis based on non-parametric tests was, therefore, appropriate. The Kruskal–Wallis test was used for comparative analysis. This test enables the comparison of three or more groups. With regard to hypothesis testing, significance was set at *p* ≤ 0.05. All analyses were conducted using IBM SPSS Statistics 25.0 for Windows.

**Figure 2:**
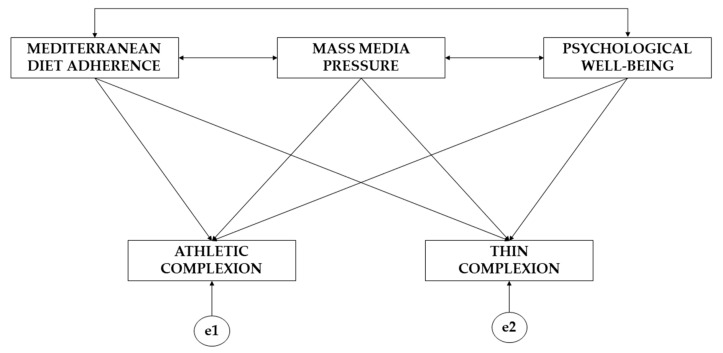
Theoretical model of the study.

In order to carry out exploratory analysis, a theoretical model was initially developed (Figure 2). In terms of the characteristics of the variables that make up this model, endogenous and exogenous variables were defined. Endogenous variables refer to all variables that are influenced by other variables [28] and act as dependent variables. In contrast, exogenous variables refer to all variables that influence other variables and do not receive the effect of any other variable [29]. The theoretical model developed consists of three exogenous variables and two endogenous variables. Due to the characteristics of the endogenous variables, an error term pertaining to the error arising from the measurement process (e1, e2) was included. Model characteristics mean that the error term pertaining to measurement error can be interpreted as a multivariate regression coefficient. With regard to the direction of arrows in the model, the structural model presents direct causal relationships, since a direct effect of the endogenous variables on the exogenous variables is outlined. A reciprocal causal relationship is not present, given that bi-directional arrows are absent from the model. Significance was set at *p* ≤ 0.05 and *p* ≤ 0.001.

Once the model was estimated, it was necessary to assess goodness of fit. The present model was evaluated using absolute fit indices (assessing residuals), relative fit indices (comparing model fit with respect to another worse fitting model) and parsimonious fit indices (assessing fit with respect to the number of parameters used) [30]. With regard to absolute fit, chi-square values and degrees of freedom were considered. In accordance with recommendations established by Maydeu-Olivares [30] and Kyriazos [31], non-significant chi-square values and degrees of freedom indicate good fit. Turning attention to fit in accordance with criteria stipulated by Maydeu-Olivares [29] and Kyriazos [31], comparative fit (CFI), Tucker–Lewis (TLI), normalized fit (NFI) and incremental fit (IFI) indices were calculated. In this case, values above 0.900 indicate good fit [32]. Examination of the root mean square residual approximation value is also recommended [28,29,30,31]. For this index, values lower than 0.100 indicate good fit [27,28,29,30,31]. Table 1 presents values produced for the different contextualized fit indices.

## 3. Results

A comparative analysis (Table 2) (Hypothesis 1 and Hypothesis 2) shows statistically significant differences (*p* ≤ 0.05) for all study variables. With regard to Mediterranean diet adherence, statistically significant differences are observed between participants who engage in less than 150 min of weekly physical activity and those who engage in more than 300 min (8.351 ± 2.193 min vs. 6.974 ± 2.737 min; *p* ≤ 0.05). Turning attention to Mediterranean diet adherence, differences were observed between participants who engaged in less than 150 min of weekly physical activity and those who engaged in between 150 and 300 min (6.974 ± 2.737 min vs. 7.862 ± 2.069 min; *p* ≤ 0.05). Participants who engaged in more than 300 min of weekly physical activity had higher levels of Mediterranean diet adherence than those who engaged in less than 300 min of weekly physical activity (8.351 ± 2.193 min vs. 7.862 ± 2.069 min vs. 6.974 ± 2.737 min). With regard to media pressure, statistically significant differences are observed between participants who engaged in less than 150 min of weekly physical activity and those who engaged in more than 300 min (2.411 ± 0.743 min vs. 2.181 ± 0.741 min; *p* ≤ 0.05). In this case, less physically active participants reported higher levels of stress than more physically active participants (2.411 ± 0.743 min vs. 2.316 ± 0.673 min vs. 2.181 ± 0.741 min). Turing attention to psychological wellbeing, statistically significant differences are observed between less active and more active participants (4.438 ± 0.703 min vs. 4.767 ± 0.586 min; *p* ≤ 0.05). More active participants were found to have higher levels of psychological wellbeing compared to all remaining participants (4.767 ± 0.586 min vs. 4.559 ± 0.794 min vs. 4.438 ± 0.703 min). With regard to slim build ideals, statistically significant differences are observed between young people who were highly active and those who are lesser active (2.935 ± 1.174 min vs. 2.616 ± 0.944 min; *p* ≤ 0.05). It is evident that less active young people feel greater pressure to achieve a slim build than other participants (2.935 ± 1.174 min vs. 2.761 ± 1.036 min vs. 2.616 ± 0.944 min). With regard to pressure to achieve an athletic build, statistically significant differences are observed between the most active and least active participants (2.513 ± 0.755 min vs. 2.086 ± 0.787 min; *p* ≤ 0.05). Finally, more active participants reported greater pressure to achieve an athletic build (2.513 ± 0.755 min vs. 2.467 ± 0.769 min vs. 2.086 ± 0.787 min).

Turning the focus to an exploratory analysis, Table 3, Table 4 and Table 5 present the outcomes as a function of weekly physical activity engagement (Hypothesis 3 and Hypothesis 4). As can be observed, within more active participants Mediterranean diet adherence (MDA) was seen to have a lesser effect on athletic complexion ideals (AC) (β = 0.080; *p* ≤ 0.05). Further, within young people who are more physically active, the effect of mass media pressure (MMP) on athletic complexion ideals (AC) (β = 0.769; *p* ≤ 0.001) was found to be more pronounced.

Turning attention to the effect of psychological wellbeing (PWB) on athletic complexion ideals (AC), negative effects are observed for both the most active (β = −0.081) and least active (β = −0.026) participants. Moving on to consider the effect of Mediterranean diet adherence (MDA) on thin complexion ideals (TC), negative relationships are observed for both the least active (β = −0.129) and most active (β = −0.071) participants. With regard to the effect of mass media pressure (MMP) on thin complexion ideals, the greatest effect (β = 0.787) was found in the most active participants. Finally, a negative effect was found between psychological wellbeing (PWB) and thin ideals in participants who engage in between 150 and 300 min of physical activity per week (β = −0.207) and in those who engage in more than 300 min of physical activity per week (β = −0.017).

## 4. Discussion

The present research reveals that participants who engage in more than 300 min of physical activity per week have better outcomes regarding Mediterranean diet adherence, wellbeing and athletic build ideals (Hypothesis 1). In contrast, less active participants report feeling greater pressure from the media to achieve a slim build. The following section discusses obtained outcomes in comparison to those reported by other similar studies (Hypothesis 2).

A comparative analysis revealed that young people who engage in more than 300 min of physical activity per week are more likely to consume a Mediterranean diet. In light of these findings, other studies have reported that young people consume an excessive amount of foods rich in saturated fats [33]. On the contrary, a subset of young people have been observed to be more concerned about their fitness [34]. This concern stems from a desire to maintain high levels of fitness and have what is deemed to be an acceptable physical image [34]. To this end, such individuals devote large amounts of time to physical conditioning and following a healthy diet [35]. In light of these findings, the need for more nutritional education in the educational sphere has been highlighted [35]. The field of study within which university students undertake academic studies has also been found to have an impact on young people’s lifestyles [36]. A study conducted by García-Pérez et al. [36] found that students undertaking sport sciences and medical sciences related courses were more active and had a healthier lifestyle.

More active participants were also observed to experience greater pressure to achieve an athletic build. This finding is not surprising given that more physically active young people are subjected to more external pressure to achieve beauty ideals dictating how their body should be which is often transmitted through social networks [37]. Studies indicate that education is needed to prevent this reality from becoming an obsession [37,38]. In cases in which striving to achieve beauty ideals becomes an obsession, specialized help is needed to address this disorder [38].

More active young people were also observed to have higher levels of emotional wellbeing. Regular weekly physical activity engagement has been shown to benefit different spheres of daily life [39]. A positive relationship has been found between psychological wellbeing and physical activity [40]. Regular physical activity engagement also helps to improve body image, which leads to greater body satisfaction and, subsequently, better psychological wellbeing [41]. Physical sports engagement has been shown to induce the secretion of neurotransmitters which act directly on emotional wellbeing [11]. Research carried out by Christensen and Galbo [42] highlighted that the type of physical activity engaged in influences wellbeing perceptions. When physical exercise is high intensity and occurs in short bouts (anaerobic), the sympathetic nervous system is activated and further neurotransmitter secretion of epinephrine and norepinephrine occurs. This leads to an increase in heart rate and respiration, activating cognitive vigilance.

With regard to media pressure, young people who engage in less than 150 min of physical activity per week report feeling more pressure. Previous research has highlighted that inactive young people report low levels of body satisfaction [43]. Dissatisfaction derives from the consumption of media that transmit impossible to reach body ideals, with such lofty ideals being highly present in many different media modalities [13]. Social networks have been revealed to be the main vehicle for transmitting ideals related to body image [13]. The achievement of the type of body image transmitted via the media has also been shown to sometimes provide a motivation for young people to engage in sport [14]. This can lead to obsessive engagement [14].

Less physically active young people were also shown to report greater pressure to achieve a slim build. In light of these findings, more sedentary individuals have been shown to have a higher body mass index and higher levels of body fat [13]. Current trends on many social networks purport body ideals that have a direct impact on young people [13]. In this case, social media currently idealizes a physical profile characterized by a slim body [44]. If this process becomes an obsession, harmful behaviors can be encouraged, such as bulimia [44].

Turning attention to exploratory outcomes, Mediterranean diet adherence was found to have a greater impact on the desire for achieving an athletic build in less active participants (Hypothesis 3). Other research studies have reported contrasting findings to those reported in the present study [13,14]. Indeed, healthy dietary patterns have been found to have a greater impact on athletic build ideals in more physically active participants [13]. In contrast to the present findings, previous research has also found greater concern about dietary care to support positive perceptions in this sphere [44]. Prior research has also observed that many young people engage in inadequate levels of physical activity due to the pressure exerted on them in the academic setting [10]. Similarly, more active individuals were found to be more susceptible to the effect of media pressure on the desire to achieve an athletic build. Generally, more active individuals tend to consume a greater amount of content related to body image and body care [43]. Such content may exert pressure on aspects related with body image [43].

The present study also revealed that within more active participants Mediterranean diet adherence had a negative impact on slim build ideals (Hypothesis 4). This may be because body ideals related to physical activity are associated with muscular body images [20]. It has also been observed that young people striving to gain muscle mass consume protein-rich foods [13], with protein being found mainly in meat [13]. In spite of this, the Mediterranean diet is characterized by a low intake of meat and a high intake of fruit and vegetables [13]. In contrast, media pressure has been found to drive individuals to strive to achieve a slim build [41]. In light of these findings, young people who follow an active and healthy lifestyle have been found to tend to strive to achieve the types of body images conveyed by the media [14]. Many young people have been observed to use these body images as motivation to engage in physical sporting activities [15].

Although the present research met its proposed research objectives, it is not without limitations. Firstly, its cross-sectional study design means that outcomes pertain to only a single point in time. Secondly, whilst the instruments used have been demonstrated to be reliable, a degree of measurement error is intrinsic to the data collection process. It should also be noted that the sample was drawn from a very specific geographical area and is not representative of the population under study. 

In terms of strengths, the present study provides a comparative and exploratory analysis in accordance with WHO standards. This made it possible to compare outcomes and examine the inter-relationships of variables via a multi-group analysis. In terms of future perspectives, experimental models should be used to examine the effects of media pressure in greater detail.

## 5. Conclusions

The main findings of the present study are that young people who engage in more than 300 min of physical activity per week show a higher level of Mediterranean diet adherence, better psychological wellbeing and experience greater pressure to achieve an athletic build. In addition, less active participants experience greater media pressure and feel more pressured to achieve a slim build. 

The exploratory analysis concludes that differential effects emerge as a function of weekly physical activity time. Specifically, more active participants are more highly susceptible to the influence of media pressure on athletic and slim build ideals. 

Finally, in the educational setting, more attention must be paid to the effects of mass media on outcomes pertaining to body image. Thus, educational programs focused on the self-acceptance of body image should be administered in the educational setting.

## Figures and Tables

**Table 1:** Multigroup model fit indices.

Absolute Fit Indices		Comparative Fit Indices				RMSEA
X^2^	Df	NFI	CFI	TLI	IFI	
2.505	2	0.996	0.998	0.975	0.998	0.045

**Table 2:** Comparative analysis as a function of weekly physical activity engagement.

		N	M ± SD	*p*
Mediterranean diet adherence	Less than 150 min	232	6.974 ± 2.737	0.033 ^a^ 0.045 ^b^
	Between 150 and 300 min	174	7.862 ± 2.069	
	More than 300 min	228	8.351 ± 2.193	
Mass media pressure	Less than 150 min	232	2.411 ± 0.743	0.017 ^a^
	Between 150 and 300 min	174	2.316 ± 0.673	
	More than 300 min	228	2.181 ± 0.741	
Psychological wellbeing	Less than 150 min	232	4.438 ± 0.703	0.049 ^a^
	Between 150 and 300 min	174	4.559 ± 0.794	
	More than 300 min	228	4.767 ± 0.586	
Thin complexion	Less than 150 min	232	2.935 ± 1.174	0.026 ^a^
	Between 150 and 300 min	174	2.761 ± 1.036	
	More than 300 min	228	2.616 ± 0.944	
Athletic complexion	Less than 150 min	232	2.086 ± 0.787	0.038 ^a^
	Between 150 and 300 min	174	2.467 ± 0.769	
	More than 300 min	228	2.513 ± 0.755	

**Note:** ^a^ Differences between participants reporting less than 150 min and more than 300 min; ^b^ differences between participants reporting less than 150 min and between 150 and 300 min; ^c^ differences between participants reporting between 150 and 300 min and more than 300 min.

**Table 3:** Standardized regression weights for participants who engage in less than 150 min of physical activity per week.

Effect Direction	Regression Weights				Standardized Regression Weights
	Estimation	Estimation Error	Critical Ratio	*p*	Estimation
AC ← MDA	0.040	0.016	2.525	≤0.05	0.140
AC ← MMP	0.712	0.064	11.122	≤0.001	0.609
AC ← PWB	−0.029	0.064	−0.452	0.651	−0.026
TC ← MDA	−0.055	0.019	−2.926	≤0.05	−0.129
TC ← MMP	1.353	0.076	17.719	≤0.001	0.775
TC ← PWB	0.172	0.077	2.249	≤0.05	0.103

**Note:** Athletic complexion ideals (AC); thin complexion ideals (TC); psychological wellbeing (PWB); mass media pressure (MMP); Mediterranean diet adherence (MDA).

**Table 4:** Standardized regression weights for participants who engage in between 150 min and 300 min of physical activity per week.

Effect Direction	Regression Weights				Standardized Regression Weights
	Estimation	Estimation Error	Critical Ratio	*p*	Estimation
AC ← MDA	0.043	0.023	1.846	0.065	0.118
AC ← MMP	0.634	0.068	9.368	≤0.001	0.624
AC ← PWB	0.111	0.064	1.720	0.085	0.116
TC ← MDA	0.067	0.023	2.874	≤0.05	0.134
TC ← MMP	1.014	0.068	15.012	≤0.001	0.727
TC ← PWB	−0.269	0.064	−4.126	≤0.001	−0.207

**Note:** Athletic complexion ideals (AC); thin complexion ideals (TC); psychological wellbeing (PWB); mass media pressure (MMP); Mediterranean diet adherence (MDA).

**Table 5:** Standardized regression weights for participants who engage in more than 300 min of physical activity per week.

Effect Direction	Regression Weights				Standardized Regression Weights
	Estimation	Estimation Error	Critical Ratio	*p*	Estimation
AC ← MDA	0.028	0.014	1.972	≤0.05	0.080
AC ← MMP	0.798	0.044	18.024	≤0.001	0.769
AC ← PWB	−0.106	0.057	−1.873	0.061	−0.081
TC ← MDA	−0.031	0.017	−1.767	0.077	−0.071
TC ← MMP	1.039	0.054	18.598	≤0.001	0.787
TC ← PWB	−0.028	0.069	−0.404	0.686	−0.017

**Note:** Athletic complexion ideals (AC); thin complexion ideals (TC); psychological wellbeing (PWB); mass media pressure (MMP); Mediterranean diet adherence (MDA).

## Data Availability

The data used to support the findings of current study are available from the corresponding author upon request.
